# Analysis of functional germline variants in *APOBEC3* and driver genes on breast cancer risk in Moroccan study population

**DOI:** 10.1186/s12885-016-2210-8

**Published:** 2016-02-26

**Authors:** Chaymaa Marouf, Stella Göhler, Miguel Inacio Da Silva Filho, Omar Hajji, Kari Hemminki, Sellama Nadifi, Asta Försti

**Affiliations:** Department of Molecular Genetic Epidemiology, German Cancer Research Center (DKFZ), Heidelberg, Germany; Laboratory of Genetics and Molecular Pathology–Medical School of Casablanca, Casablanca, Morocco; University Hassan II Ain Chock, Center Of Doctoral Sciences “In Health Sciences”, Casablanca, Morocco; Department of Oncology, Littoral Clinic, Casablanca, Morocco; Center for Primary Health Care Research, Clinical Research Center, Lund University, Malmö, Sweden

**Keywords:** Breast cancer, Driver genes, *APOBEC3*, Genetic susceptibility, Single nucleotide polymorphism

## Abstract

**Background:**

Breast cancer (BC) is the most prevalent cancer in women and a major public health problem in Morocco. Several Moroccan studies have focused on studying this disease, but more are needed, especially at the genetic and molecular levels. Therefore, we investigated the potential association of several functional germline variants in the genes commonly mutated in sporadic breast cancer.

**Methods:**

In this case–control study, we examined 36 single nucleotide polymorphisms (SNPs) in 13 genes (*APOBEC3A, APOBEC3B, ARID1B, ATR, MAP3K1, MLL2, MLL3, NCOR1, RUNX1, SF3B1, SMAD4, TBX3, TTN*), which were located in the core promoter, 5’-and 3’UTR or which were nonsynonymous SNPs to assess their potential association with inherited predisposition to breast cancer development. Additionally, we identified a ~29.5-kb deletion polymorphism between *APOBEC3A* and *APOBEC3B* and explored possible associations with BC. A total of 226 Moroccan breast cancer cases and 200 matched healthy controls were included in this study.

**Results:**

The analysis showed that12 SNPs in 8 driver genes, 4 SNPs in *APOBEC3B* gene and 1 SNP in *APOBEC3A* gene were associated with BC risk and/or clinical outcome at *P ≤ 0.05* level. *RUNX1*_rs8130963 (odds ratio (OR) = 2.25; 95 % CI 1.42-3.56; *P* = 0.0005; dominant model), *TBX3*_rs8853 (OR = 2.04; 95 % CI 1.38-3.01; *P* = 0.0003; dominant model), *TBX3*_rs1061651 (OR = 2.14; 95 % CI1.43-3.18; *P* = 0.0002; dominant model), *TTN*_rs12465459 (OR = 2.02; 95 % confidence interval 1.33-3.07; *P* = 0.0009; dominant model), were the most significantly associated SNPs with BC risk. A strong association with clinical outcome were detected for the genes *SMAD4* _rs3819122 with tumor size (OR = 0.45; 95 % CI 0.25-0.82; *P* = 0.009) and *TTN*_rs2244492 with estrogen receptor (OR = 0.45; 95 % CI 0.25-0.82; *P* = 0.009).

**Conclusion:**

Our results suggest that genetic variations in driver and *APOBEC3* genes were associated with the risk of BC and may have impact on clinical outcome. However, the reported association between the deletion polymorphism and BC risk was not confirmed in the Moroccan population. These preliminary findings require replication in larger studies.

## Background

Breast Cancer (BC) is one of the most frequent malignant disease and primary cause of death in women worldwide. Approximately 522,000 women died on BC in 2012 and 1.67 million new cancer cases were diagnosed worldwide [1, 2].

The vast majority of sporadic and familial breast cancer cases arise due to lifelong accumulation of genetic factors in the breast tissue. Recent genome-wide association studies (GWASs) focusing on evaluating common single nucleotide polymorphisms (SNPs) have identified more than 70 genetic susceptibility loci for breast cancer [3–25]. Partial and full tumor genome sequences have revealed the existence of hundreds to thousands of mutations in most cancers [26–32]. However, genome sequencing has revealed that many cancers, including breast cancer, have somatic mutation spectra dominated by C-to-T transitions [27–32]. Recently, the International Cancer Genome Consortium (ICGC) was launched to identify those somatic mutations and consequently to determine those genes which are required for human cancer development [29, 33]. Approximately 10 % of those are driver mutations, which initiate the carcinogenic process [34].

Additionally, recent studies have shown that copy number variations (CNVs), another type of genetic variation, occur frequently in the genome and account for more nucleotide sequence variation than single-nucleotide polymorphisms [35]. This variation accounts for roughly 12 % of human genomic DNA, and each variation may range from about 1 kb to several megabases in size [36]. Recently, through CNV GWAS, Long et al. [37] discovered a common CNV locus for breast cancer in Chinese women, which was located between exon 5 of *APOBEC3A* and exon 8 of *APOBEC3B*, resulting in a fusion gene with a protein sequence identical to *APOBEC3A*, but with a 3’-UTR of *APOBEC3B*. This deletion has been associated with increased BC risk in both Chinese and a Caucasian population with a population frequency of around 37 and 6 % respectively [37–39]. In addition to decreased expression of *APOBEC3B*, the deletion may lead to alteration in *APOBEC3A* RNA stability.

Considering the potential function of driver and *APOBEC3* gene in the process of tumorigenesis in BC, it is possible that germline variations and CNV in those genes could influence the risk of BC. For this reason, we conducted this case–control study in a sample of Moroccan women.

## Methods

### Study population

The present case–control study was performed involving 226 cases, recruited from the Department of Oncology of the Littoral Clinic of Casablanca during 2013. The control group included a total of 200 healthy women with no personal history of cancer diseases selected from DNA bank volunteers of the Genetics and Molecular Pathology Laboratory. Clinico-pathological parameters including age at diagnosis, menopausal status, histology type, tumor size, Scarff-Bloom-Richardson (SBR) grade, lymph nodes status, and hormone receptors status were obtained from patients’ medical records. The study protocols have been approved by the Ethic Committee for Biomedical Research in Casablanca (CERBC) of the Faculty of Medicine and Pharmacy and written informed consent was obtained from each subject.

### Gene/SNP selection

Regarding driver genes, we focused on genes described to carry BC driver mutations in at least two of the following publications: Stephens et al. 2012; Banerji et al. 2012; Ellis et al. 2012; Shah et al. 2012 [32, 40–42]. The well-known and intensively studied genes such as *BRCA1* or *PTEN* were excluded from this study. A total of 36 SNPs across 11 driver genes (*ARID1B, ATR, MAP3K1, MLL2, MLL3, NCOR1, RUNX1, SF3B1, SMAD4, TBX3, TTN*) and 2 genes of *APOBEC3* family (*APOBEC3A, APOBEC3B*) were selected to the study based on data obtained from Ensembl Genome browser (http://www.ensembl.org/index.html) for the CEU (Utah residents with Northern and Western European ancestry from the CEPH collection). The SNPs selection was based on these criteria: (1) minor allele frequency (MAF) value over 10 %; (2) location within the coding region (non synonymous SNPs), core promoter regions and 5’- and 3’-untranslated regions (UTRs), (3) Haploview was used to select SNPs on the basis of linkage disequilibrium (LD; r^2^ ≥ 0.80)) to minimize the number of SNPs to be genotyped. RegulomeDB (http://www.regulomedb.org/) was used to explore the potential function of the associated SNPs.

### Genotyping

Genomic DNA was extracted from peripheral blood leukocytes using the salting out procedure [31]. Genomic DNA was dissolved in TE (10 mM Tris–HCl and 0.1 mM EDTA, pH8.0). Spectrophotometry was used to quantify DNA using the Nanovue TM Plus spectrophotometer.

Genotyping was performed using TaqMan SNP Genotyping Assay from Life Technologies (Darmstadt, Germany) or KASPar SNP Genotyping system from KBioscience (Hoddesdon, Great Britain) in a 384-well plate format. Master Mix for the the KASPar assay was prepared according to the KBioscience’s conditions and products, whereas 5× HOT FIREPol Probe qPCR Mix Plus from Solis BioDyne (Tartu, Estonia) for TaqMan SNP Genotyping Assay was used. The Polymerase chain reactions (PCR) were performed in a final reaction volume of 5 μl per well. The PCR poducts were analyzed using ViiA7 Real-Time PCR System from Applied Biosystems (Weiterstadt, Germany).

### *Screening for APOBEC3* deletion

Polymerase chain reaction (PCR) was carried out to amplify *APOBEC3* gene in a final volume of 10 μl containing 10× reaction buffer, 50 mM MgCl_2_, 10 mM dNTPs, 10 μM primers, 5U Taq DNA polymerase, and 10 ng genomic DNA. The PCR amplification parameters were 40 cycles of 1 min of denaturing at 95 °C, 1 min of annealing at 60 °C, and 1 min of extension at 72 °C.

The insertion and deletion alleles were detected by amplifying genomic DNA with the following oligonucleotide sequences:

Deletion_F:TAGGTGCCACCCCGAT;Deletion_R:TTGAGCATAATCTTACTCTTGTAC; Insertion1_F: TTGGTGCTGCCCCCTC; Insertion1_R: TAGAGACTGAGGCCCAT; and Insertion2_F: TGTCCCTTTTCAGAGTTTGAGTA; Insertion2_R: TGGAGCCAATTAATCACTTCAT. Deletion alleles resulted in 700 bp fragment, Insertion1alleles resulted in 490 bp fragment and Insertion2 alleles resulted in 705 bp fragment. Insertion and deletion PCR assays were performed separately, the products pooled, and visualized by ethidium bromide staining on a standard 1.5 % agarose gel.

### Statistical analysis

The Hardy Weinberg equilibrium (HWE) was tested by comparing observed and expected genotype frequencies in both cases and controls using *χ*2 test. Odds ratio with a confidence intervals (CIs) of 95 % were calculated using multiple logistic regression (PROC LOGISTIC, SAS Version 9.2; SAS Institute, Cary, NC) to assess the strength of the association between genotypes and breast cancer risk. The *P value* ≤ 0.05 was considered statistically significant.

### In Silico prediction

To investigate how the SNPs can influence the gene expression and their consequences on protein binding sites, chromatin structure and promoter and enhancer strength, we used HaploReg (http://www.broadinstitute.org/mammals/haploreg/haploreg.php). To identify the possible effects on histone modification we used RegulomeDB (http://regulome.stanford.edu/). These effects were proofed for data in MCF7 (Michigan Cancer Foundation-7 breast cancer cell line), T-47D (epithelial cell line derived from mammary ductal carcinoma), HMEC (human mammary epithelial cells) or MCF10A-ER-SRc (breast epithelial cell line -estrogen receptor –src) cell lines. SIFT and PolyPhen predictions were used to determine the possible effect of amino acid substitutions on protein function and structure (Ensemble release 75, http://www.ensembl.org/index.html). The MicroSNiPer was used to predict the impact of all the significant SNPs of this study located in 3’UTR on micro-RNA binding using microSNiPer (http://epicenter.ie-freiburg.mpg.de/services/microsniper/).

## Results

The baseline characteristics of the population sample analyzed in our study are listed in Table 1. In total, 226 BC cases and 200 controls were successfully genotyped for 36 selected SNPs in 13 potential genes. Altogether 12 SNPs in 8 driver genes, 4 SNPs in *APOBEC3B* gene and 1 SNP in *APOBEC3A* gene were associated with BC risk and/or clinical outcome at *P ≤ 0.05* level (Tables 2 and 3).

**Table 1:** Characteristics of breast tumors at time of diagnosis

Characteristics	Samples
Cases/Controls	226/200
Age at diagnosis, mean ± SD (years)	41 ± 11
Range (years)	27 – 67
Menopausal Status	No. (%)
Premenopausal	162(71.68)
Postmenopausal	63(27.87)
Missing	1(0.44)
Estrogen receptor	
Positive	130 (57.52)
Negative	78(34.51)
Missing	18 (7.96)
Progesterone receptor	
Positive	136 (59.29)
Negative	72(31.85)
Missing	18 (7.96)
Estrogen/Progesterone receptor	
ER^+^/PR^+^	111 (49.11)
ER^+^/PR^−^	25 (11.06)
ER^−^/PR^+^	19 (8.40)
ER^−^/PR^−^	53 (23.45)
Tumor size	
<2 cm	30 (13.27)
>2 cm	105 (46.46)
>5 cm	41(18.14)
Tumor of any size with extension	37 (16.37)
Histological grade	
1	8 (3.53)
2	141 (62.38)
3	59 (26.10)
Lymph node status	
Negative	86(38.55)
Positive	132 (58.40)
Distant metastases	
Negative	170(75.22)
Positive	38 (16.81)

ER estrogen receptors, PR progesterone receptors

**Table 2:** SNPs associated with breast cancer risk

Breast cancer risk					
Gene/SNP	Genotype	Cases (%)	Controls (%)	OR (95 % CI)	*P*-value
*APOBEC3B*	CC	181 (80.09)	176 (88.00)	1.00	
rs8142462	TC	42 (18.58)	24 (12.00)	1.70 (0.99-2.93)	0.0500
	TT	3 (1.33)	0 (0.00)	0 (0)	0.9839
	Dom	45 (19.91)	24 (12.00)	1.82 (1.07-3.12)	0.0300
	Overall				0.1584
*APOBEC3A*	GG	111 (49.12)	125 (62.50)	1.00	
rs17370615	GA	102 (45.13)	66 (33.00)	1.74 (1.16-2.60)	0.0068
	AA	13 (5.75)	9 (4.50)	1.63 (0.67-3.95)	0.2826
	Dom	115 (50.88)	75 (37.50)	1.73 (1.17-2.54)	0.0050
	Overall				0.0217
*APOBEC3B*	CC	95 (42.0)	69 (34.50)	1.00	
rs28401571	CT	93 (41.15)	80 (40.00)	0.84 (0.55-1.30)	0.4412
	TT	38 (16.81)	51 (25.50)	0.54 (0.32-0.91)	0.0212
	Add			0.75 (0.58-0.97)	0.0300
	Overall				0.0682
*APOBEC3B*	TT	82 (36.28)	93 (46.50)	1.00	
rs6001376	CT	106 (46.90)	87 (43.50)	1.38 (0.92-2.08)	0.1226
	CC	38 (16.81)	20 (10.00)	2.15 (1.16-4.00)	0.0148
	Add			1.44 (1.09-1.91)	0.0100
	Overall				0.0390
*APOBEC3B*	CC	44 (19.47)	49 (24.50)	1.00	
rs1065184	CT	128 (56.64)	119 (59.50)	1.20 (0.74-1.93)	0.4587
	TT	54 (23.89)	32 (16.00)	1.88 (1.03-3.42)	0.0385
	Add			1.36 (1.01-1.84)	0.0400
	Overall				0.1000
*ATR*	GG	78 (34.51)	94(47.00)	1.00	
rs2227928	AG	110 (48.67)	87(43.50)	1.52 (1.01-2.30)	0.0448
	AA	38 (16.81)	19(9.50)	2.41 (1.29-4.51)	0.0060
	AG + AA	148 (65.49)	106(53.00)	1.68 (1.14-2.49)	0.0090
	Overall				0.0123
*ARID1B*	CC	50 (22.12)	63 (31.50)	1.00	
rs73013281	CT	126 (55.75)	90 (45.00)	1.76 (1.11-2.79)	0.0154
	TT	50 (22.12)	47 (23.50)	1.34 (0.78-2.31)	0.2915
	CT + TT	176 (77.88)	137 (68.50)	1.62 (1.05-2.50)	0.0293
	Overall				0.0500
*MAP3K1*	CC	130 (57.52)	137 (68.50)	1.00	
rs832583	AC	80 (35.40)	58 (29.00)	1.45 (0.96-2.20)	0.0770
	AA	16 (7.08)	5 (2.50)	3.37 (1.20-9.47)	0.0210
	AC + CC	96 (42.48)	63 (31.50)	1.61 (1.08-2.39)	0.0197
	Overall				0.0236
*NCOR1*	CC	102 (45.13)	108 (54.00)	1.00	
rs178831	CT	103 (45.58)	82 (41.00)	1.33 (0.89-1.98)	0.1589
	TT	21 (9.29)	10 (5.00)	2.22 (1.00-4.95)	0.0500
	CT + TT	124 (54.87)	92 (46.00)	1.43 (0.97-2.09)	0.0681
	Overall				0.0908
*RUNX1*	AA	153 (67.70)	165 (82.50)	1.00	
rs8130963	AG	70 (30.97)	33 (16.50)	2.29 (1.43-3.65)	0.0005
	GG	3 (1.33)	2 (1.00)	1.62 (0.27-9.81)	0.6010
	AG + GG	73 (32.30)	35 (17.50)	2.25 (1.42-3.56)	0.0005
	Overall				0.0024
*RUNX1*	CC	53 (23.45)	71 (35.50)	1.00	
rs17227210	CT	123 (54.42)	92 (46.00)	1.79 (1.15-2.80)	0.0106
	TT	50 (22.12)	37 (18.50)	1.81 (1.04-3.15)	0.0359
	CT + TT	173 (76.55)	129 (64.50)	1.80 (1.18-2.74)	0.0066
	Overall				0.0249
*SMAD4*	AA	145 (64.16)	157 (78.50)	1.00	
rs12456284	AG	72 (31.86)	39 (19.50)	2.00 (1.27-3.14)	0.0026
	GG	9 (3.98)	4 (2.00)	2.44 (0.73-8.08)	0.1457
	AG + GG	81 (35.84)	43 (21.50)	2.04 (1.32-3.15)	0.0013
	Overall				0.0053
*TBX3*	CC	104 (46.02)	127 (63.50)	1.00	
rs8853	CT	106 (46.90)	60 (30.00)	2.16 (1.43-3.25)	0.0002
	TT	16 (7.08)	13 (6.50)	1.50 (0.69-3.27)	0.3037
	CT + TT	122 (53.98)	73 (36.50)	2.04 (1.38-3.01)	0.0003
	Overall				0.0011
*TBX3*	TT	118 (52.21)	140 (70.00)	1.00	
rs1061651	TC	97 (42.92)	50 (25.00)	2.30 (1.51-3.50)	0.0001
	CC	11 (4.87)	10 (5.00)	1.31 (0.54-3.18)	0.5579
	TC + CC	108 (47.79)	60 (30.00)	2.14 (1.43-3.18)	0.0002
	Overall				0.0005
*TBX3*	GG	89 (39.38)	106 (53.00)	1.00	
rs2242442	AG	104 (46.02)	84 (42.00)	1.47 (0.99-2.21)	0.0500
	AA	33 (14.60)	10 (5.00)	3.93 (1.84-8.42)	0.0004
	AG + AA	137 (60.62)	94 (47.00)	1.74 (1.18-2.55)	0.0050
	Overall				0.0012
*TTN*	AA	131 (57.96)	139(69.50)	1.00	
rs12463674	AG	85 (37.61)	53(26.50)	1.70 (1.12-2.58)	0.0127
	GG	10 (4.42)	8(4.00)	1.33 (0.51-3.46)	0.5641
	AG + GG	95 (42.04)	61(30.50)	1.65 (1.11-2.47)	0.0140
	Overall				0.0436
*TTN*	CC	135 (59.73)	150 (75.00)	1.00	
rs12465459	CT	84 (37.17)	46 (23.00)	2.03 (1.32-3.11)	0.0012
	TT	7 (3.10)	4 (2.00)	1.94 (0.56-6.79)	0.2972
	CT + TT	91 (40.27)	50 (25.00)	2.02 (1.33-3.07)	0.0009
	Overall				0.0041

OR odds ratio, CI confidence interval, SNP single nucleotide polymorphism

**Table 3:** SNPs associated with clinico-pathological features

Gene/SNP	Genotype	Significant association	No. of patients Group 1(%)	No. of patients Group 2(%)	OR (95 % CI)	*P*-value	Significant association	No. of patients Group 1(%)	No. of patients Group 2(%)	OR (95 % CI)	*P-*value
*APOBEC3B*		Tumor size	≤2 cm	>2 cm							
rs8142462	CC		68 (87.18)	105 (76.09)	1.00						
	TC		8 (10.26)	32 (23.19)	2.59 (1.13-5.96)	0.0300					
	TT		2 (2.56)	1 (0.72)	0.32 (0.03-3.64)	0.3600					
	TC + TT		10 (12.82)	33 (23.91)	2.14 (0.99-4.62)	0.0500					
	Overall					0.0500					
*APOBEC3B*		Estrogen receptor/Progesterone receptors	ER+/PR+	ER-/PR-			Estrogen receptor	ER+	ER-		
rs28401571	CC		48 (43.24)	21 (39.62)	1.00			59 (43.38)	30 (41.67)	1.00	
	CT		49 (44.14)	16 (30.19)	0.75 (0.35-1.60)	0.4500		62 (45.59)	22 (30.56)	0.70 (0.36-1.34)	0.2800
	TT		14 (12.61)	16 (30.19)	2.61 (1.08-6.31)	0.0300		15 (11.03)	20 (27.78)	2.62 (1.18-5.84)	0.0200
	CT + TT		63 (56.76)	32 (60.38)	1.16 (0.60-2.26)	0.6600		77 (56.62)	42 (58.33)	1.07 (0.60-1.91)	0.8100
	Overall					0.0200					0.0100
*APOBEC3B*		Estrogen receptor/Progesterone receptors	ER+/PR+	ER-/PR-							
rs2076111	CC		40 (36.04)	11 (20.75)	1.00						
	CT		67 (60.36)	41 (77.36)	2.23 (1.03-4.82)	0.0400					
	TT		4 (3.60)	1 (1.89)	0.91 (0.09-8.98)	0.9300					
	CT + TT		71 (63.96)	42 (79.25)	2.15(1.00-4.64)	0.0500					
	Overall					0.1000					
*ATR*		Tumor Size	≤2 cm	>2 cm			Estrogen receptor/Progesterone receptors	ER+/PR+	ER+/PR-		
rs2227928	GG		33 (42.31)	40 (28.99)	1.00			33 (29.73)	13 (52.00)	1.00	
	AG		34 (43.59)	71 (51.45)	1.72 (0.93-3.19)	0.0800		58 (52.25)	10 (40.00)	0.44 (0.17-1.11)	0.0800
	AA		11 (14.10)	27 (19.57)	2.02 (0.88-4.69)	0.0900		20 (18.02)	2 (8.00)	0.25 (0.05-1.24)	0.0900
	AG + AA		45 (57.69)	98 (71.01)	1.80 (1.01-3.21)	0.0400		78 (70.27)	12 (48.00)	0.39 (0.16-0.95)	0.0300
	Overall					0.1300					0.0900
*MLL2*		Tumor Size	≤2 cm	>2 cm			Histologic grade	1 + 2	3		
rs11614738	GG		26 (33.33)	61 (44.20)	1.00			18 (30.51)	69 (46.31)	1.00	
	CG		37 (47.44)	64 (46.38)	0.74 (0.40-1.36)	0.3200		35 (59.32)	59 (39.60)	0.44 (0.23-0.86)	0.0100
	CC		15 (19.23)	13 (9.42)	0.37 (0.15-0.88)	0.0200		6 (10.17)	21 (14.09)	0.91 (0.32-2.60)	0.8600
	CG + CC		52 (66.67)	77 (55.80)	0.63 (0.35-1.13)	0.1100		41 (69.49)	80 (53.69)	0.51 (0.27-0.97)	0.0300
	Overall					0.0800					0.0300
*SMAD4*		Histologic grade	1 + 2	3							
rs12456284	AA		36 (61.02)	99 (66.44)	1.00						
	AG		18 (30.51)	47 (31.54)	0.95 (0.49-1.84)	0.8700					
	GG		5 (8.47)	3 (2.01)	0.22 (0.05-0.96)	0.0400					
	AG + GG		23 (38.98)	50 (33.56)	0.79 (0.42-1.48)	0.4600					
	Overall					0.1300					
*SMAD4*		Tumor Size	≤2 cm	>2 cm			Estrogen receptor/Progesterone receptors	ER+/PR+	ER+/PR-		
rs3819122	AA		22 (28.21)	64 (46.38)	1.00			43 (38.74)	15 (60.00)	1.00	
	AC		45 (57.69)	52 (37.68)	0.40 (0.21-0.74)	0.0030		48 (43.24)	7 (28.00)	0.42 (0.16-1.12)	0.0800
	CC		11 (14.10)	22 (15.94)	0.69 (0.29-1.64)	0.3900		20 (18.02)	3 (12.00)	0.43 (0.11-1.66)	0.2100
	AC + CC		56 (71.79)	74 (53.62)	0.45 (0.25-0.82)	0.0090		68 (61.26)	10 (40.00)	0.42 (0.17-1.02)	0.0500
	Overall					0.0100					0.1600
*TBX3*		Histologic grade	1 + 2	3							
rs3759173	GG		11 (18.64)	47 (31.54)	1.00						
	GT		34 (57.63)	69 (46.31)	0.47 (0.22-1.03)	0.0500					
	TT		14 (23.73)	33 (22.15)	0.55 (0.22-1.37)	0.1900					
	GT + TT		48 (81.36)	102 (68.46)	0.50 (0.24-1.04)	0.0600					
	Overall					0.1600					
*TBX3*		Regional lymph node met	N-	N+							
rs8853	CC		67 (50.76)	33 (38.37)	1.00						
	CT		53 (40.15)	49 (56.98)	1.88 (1.06-3.32)	0.0300					
	TT		12 (9.09)	4 (4.65)	0.68 (0.20-2.26)	0.5200					
	CT + TT		65 (49.24)	53 (61.63)	1.66 (0.95-2.88)	0.0700					
	Overall					0.0400					
*TTN*		Regional lymph node met	N-	N+							
rs2303838	CC		87 (65.91)	50 (58.14)	1.00						
	CT		42 (31.82)	29 (33.72)	1.20 (0.67-2.16)	0.5400					
	TT		3 (2.27)	7 (8.14)	4.06 (1.00-16.4)	0.0400					
	CT + TT		45 (34.09)	36 (41.86)	1.39 (0.80-2.44)	0.2400					
	Overall					0.1300					
*TTN*		Estrogen receptor	ER+	ER-			Estrogen receptor/Progesterone receptors	ER+/PR+	ER-/PR-		
rs2244492	CC		36 (26.47)	32 (44.44)	1.00			31 (27.93)	23 (43.40)	1.00	
	CT		77 (56.62)	32 (44.44)	0.47 (0.25-0.88)	0.0100		63 (56.76)	25 (47.17)	0.53 (0.26-1.09)	0.0800
	TT		23 (16.91)	8 (11.11)	0.39 (0.15-1.00)	0.0400		17 (15.32)	5 (9.43)	0.40 (0.13-1.23)	0.1000
	CT + TT		100 (73.53)	40 (55.56)	0.45 (0.25-0.82)	0.0090		80 (72.07)	30 (56.60)	0.51 (0.26-1.00)	0.0500
	Overall					0.0300					0.1300
*TTN*		Progesterone receptor	PR+	PR-			Estrogen receptor/Progesterone receptors	ER+/PR+	ER-/PR-		
rs12465459	CC		87 (66.92)	40 (51.28)	1.00			74 (66.67)	27 (50.94)	1.00	
	CT		39 (30.00)	36 (46.15)	2.01 (1.12-3.61)	0.0200		34 (30.63)	24 (45.28)	1.93 (0.98-3.83)	0.0500
	TT		4 (3.08)	2 (2.56)	1.09 (0.19-6.18)	0.9200		3 (2.70)	2 (3.77)	1.83 (0.29-11.54)	0.5200
	CT + TT		43 (33.08)	38 (48.72)	1.92 (1.08-3.42)	0.0200		37 (33.33)	26 (49.06)	1.93 (0.99-3.75)	0.0500
	Overall					0.0600					0.1500
*TTN*		Progesterone receptor	PR+	PR-			Regional lymph node met	N-	N+		
rs12463674	AA		70 (53.85)	51 (65.38)	1.00			71 (53.79)	56 (65.12)	1.00	
	AG		56 (43.08)	22 (28.21)	0.54 (0.29-0.99)	0.0400		56 (42.42)	25 (29.07)	0.57 (0.31-1.02)	0.0500
	GG		4 (3.08)	5 (6.41)	1.72 (0.44-6.71)	0.4300		5 (3.79)	5 (5.81)	1.27 (0.35-4.60)	0.7100
	AG + GG		60 (46.15)	27 (34.62)	0.62 (0.35-1.10)	0.1000		61 (46.21)	30 (34.88)	0.62 (0.36-1.09)	0.0900
	Overall					0.0700					0.1300
		Histologic grade	1 + 2	3			Estrogen receptor/Progesterone receptors	ER+/PR+	ER-/PR+		
			34 (57.63)	88 (59.06)	1.00			64 (57.66)	6 (31.58)	1.00	
			19 (32.20)	58 (38.93)	1.18 (0.61-2.26)	0.6100		44 (39.64)	12 (63.16)	2.91 (1.02-8.33)	0.0400
			6 (10.17)	3 (2.01)	0.19 0.05-0.82)	0.0200		3 (2.70)	1 (5.26)	3.56 (0.32-39.70)	0.3000
			25 (42.37)	61 (40.94)	0.94 (0.51-1.74)	0.8400		47 (42.34)	13 (68.42)	2.95 (1.04-8.33)	0.0400
						0.0500					0.1200

OR odds ratio, CI confidence interval, SNP single nucleotide polymorphism, No total number

The most significant associations with BC risk were observed for *RUNX1*_rs8130963 (OR = 2.25; 95 % CI 1.42-3.56; *P* = 0.0005; dominant model), *TBX3*_rs8853 (OR = 2.04; 95 % CI 1.38-3.01; *P* = 0.0003; dominant model), *TBX3*_rs1061651 (OR = 2.14; 95 % CI 1.43-3.18; *P* = 0.0002; dominant model), *TTN*_rs12465459 (OR = 2.02; 95 % CI 1.33-3.07; *P* = 0.0009; dominant model). However, the strongest significant associations were observed for *TBX3*_rs2242442, *ATR*_rs2227928, *RUNX1*_rs17227210; both heterozygous and homozygous carriers of the minor allele were at increased risk of BC (Table 2). Considering driver gene, only the SNP rs2227928 in *ATR* was associated both with risk (OR 1.68, 95 % CI 1.14-2.49 dominant model), tumor size and hormone receptor status (Table 3).

An increased risk was observed for homozygous carriers of the minor allele for rs178831 in *NCOR1* (OR 2.22, 95%CI 1.00-4.95) (Table 2), however no association with clinical tumor characteristics was observed. Two of the six genotyped SNPs in *TTN* were associated with less aggressive tumor features: rs12463674 with low histological grade and rs2244492 with low hormone receptor status (Table 3). Additionally, the minor allele carriers of the SNPs rs6001376 in *APOBEC3B* and rs832583 in *MAP3K1* had an increased risk of BC (OR 2.15, 95 % CI 1.16-4.00; OR and OR 3.37, 95 % CI 1.20-9.47, respectively) (Table 2). Three additional SNPs in *APOBEC3B* showed associations with clinic-pathological features: large tumor size and hormone receptor status (Table 3). An increased risk was observed for rs12456284 in *SMAD4*(OR 2.04, 95%CI 1.32-3.15). The SNP was also associated with histologic grade. No correlation was observed between *APOBEC3* deletion and clinic-pathological parameters of breast cancer either in the hormone receptor status, tumor size, histological grade, lymph node status and distant metastases (Table 4). In addition, no statistically significant association was observed between *APOBEC3* deletion and breast cancer risk (Table 5).

**Table 4:** Frequencies of *APOBEC3* deletion according to clinic-pathological features

	*APOBEC3* deletion	
Variable	II	ID
Estrogen/Progesterone receptor	No. (%)	No. (%)
ER^+^/PR^+^	103 (45.57)	8 (3.53)
ER^+^/PR^−^	21 (9.29)	4 (1.76)
ER^−^/PR^+^	18(7.96)	1 (0.44)
ER^−^/PR^−^	50(22.12)	3 (1.32)
Tumor size		
<2 cm	26 (11.50)	4 (1.76)
>2 cm	97 (42.92)	8 (3.53)
>5 cm	39 (17.25)	2 (0.88)
Tumor of any size with extension	32 (14.15)	5 (2.21)
Histological grade		
1	7 (3.09)	1 (0.44)
2	127 (56.19)	14 (6.19)
3	56 (24.77)	3 (1.32)
Lymph node status		
Negative	64 (28.31)	8 (3.53)
Positive		
	122 (53.98)	
	10 (4.42)	
Distant metastases		
Negative	158 (69.91)	12 (5.30)
Positive		
	31 (13.71)	7 (3.09)

II homozygous insertion, ID herozygous deletion, No total number, ER estrogen receptors, PR progesterone receptors

**Table 5:** Genotype of *APOBEC3* deletion polymorphism in breast cancer patients and healthy controls

Breast cancer risk				
Genotype	Cases (%)	Controls (%)	OR (95 % CI)	*P*-value
II	207 (91.59)	175 (87.50)	1.00	
ID	19 (8.41)	25 (12.50)	0.64 (0.34-1.21)	0.1680
DD	0 (0)	0 (0)	0 (0)	
ID + DD	19 (8.41)	25 (12.50)	0.64 (0.34-1.21)	0.1680
Overall				0.1680

II homozygous insertion, ID herozygous deletion, DD homozygous deletion, No total number, OR odds ratio, CI confidence interval

## Discussion

In this population-based case–control study, we investigated for the first time the influence of the germline variation and CNVs in the potential driver genes and *APOBEC3* genes on breast cancer susceptibility in a North African population.

The *APOBEC3* genes family, including *APOBEC3A, APOBEC3B, APOBEC3C, APOBEC3D*, *APOBEC3E, APOBEC3F, APOBEC3G*, and *APOBEC3H*, plays pivotal roles in intracellular defense against viral infections [43]. The *APOBEC3* genes family encodes cytosine deaminases that have been implicated in innate immune responses by restricting retroviruses, mobile genetic elements like retro-transposons and endogenous retroviruses [44]. Furthermore, the *APOBEC3* genes may play a role in carcinogenesis by triggering DNA mutation through dC deamination [45]. Moreover, expression of the *APOBEC3* genes is regulated by estrogen [46], a hormone that plays a central role in the etiology of breast cancer. Very recently, Burns et al. provided evidence that *APOBEC3B* is overexpressed in breast cancer tumors and cell lines and that the *APOBEC3B* mutation signature is statistically more prevalent in the breast tumor database of The Cancer Genome Atlas (TCGA) than is expected [47]. Interestingly, the *APOBEC3B* mutation signature was detectable in colorectal and prostate cancers only when whole- genome, but not whole-exome, data were used, suggesting a tissue-specific bias against enrichment of mutations by *APOBEC3B* in coding regions. Both studies from Burns et al. and Roberts et al. reached the same conclusion that the *APOBEC3B* mutation signature is specifically enriched in six types of cancers, including those of the cervix, bladder, lung (adeno and squamous cell), head and neck, and breast [47, 48].

Furthermore, the *APOBEC3* deletion is 29.5 kb in length, located between exon 5 of *APOBEC3A* gene and exon 8 of *APOBEC3B* gene resulting in complete removal of the coding region of the *APOBEC3B* gene. This deletion is associated with decreased expression of the *APOBEC3B* gene in breast cancer cells [46]. Somatic deletion of this 29.5 kb has also been observed in breast and oral cancer tumor tissue [39, 46]. In the present study, our results did not reveal significant association between *APOBEC3* deletion polymorphism and breast cancer risk (Table 5). This result is in agreement with a Japanese case–control study of 50 cases and 50 controls reporting a non-statistically significant risk of breast cancer associated with homozygous deletion of this region (OR = 3.91, 95 % CI = 0.77 to 19.83) [49]. Nevertheless, there are some studies showing an important role of this CNVs in breast cancer and provide additional evidence to implicate *APOBEC3* deletion as a novel susceptibility factor for breast cancer risk [37, 39].

In addition, our genetic data pointed to the possible involvement of genetic variants within the studied genes *NCOR1, RUNX1, SMAD4, TBX3, TTN, ATR, ARID1B* and *MAP3K1.* The most significant association with breast cancer risk was identified by *RUNX1*_rs8130963, *RUNX1*_ rs17227210, *TBX3*_rs8853, *TBX3*_ rs1061651, *TBX3*_2242442, TTN_rs12463674, and *ATR*_rs2227928. The other driver gene did not reveal an important role in breast cancer risk.

*RUNX1* (Run-Related Transcription Factor 1) also known as *AML1* (acute myeloid leukemia 1 gene) is a tumor suppressor gene with a length of 1,196,949 bp and was original identified in acute myeloid leukemia (AML). Previously, several studies have suggested that the *RUNX1* gene is highly expressed in breast epithelial cells and it is frequently mutated in breast cancer [50]. Down regulation of *RUNX1* is part of a 17-gene signature that has been suggested to predict breast cancer metastasis [51]. In the present study, 2 of 3 genotyped SNPs (rs8130963 and rs17227210) were associated with breast cancer risk. Rs8130963 shows a strong genetic differentiation between the European and African population (Fst = 0.346), which is an indication for positive selection. Interestingly rs17227231 which is linked with an r^2^ = 92 to rs17227210 could change the protein binding of GATA3 (GATA binding protein3) as well as the transcription factor binding site of GATA. GATA3 was already classified as a high confident driver gene for breast [52]. On the other hand, rs17227210 has an effect in splicing. The variant C do not bind SF2/ASF which is involved in alternative mRNA splicing. It is a member of the serine/arginine rich protein family and was found to be up regulated in diverse tumors [49].

The T-box transcription factor 3 (13,910 bp) is expressed in mammary tissues and plays therefore a context-dependent role in mammary gland development as well as in mammary tumor genesis [53]. In addition, The *TBX3* is overexpressed in a number of breast cancer cell lines [54] and could serve as a biomarker [55]. Our results reveal that one of genotyped SNPs in *TBX3* was associated both with breast cancer risk and clinical outcome. Rs8853 apparently has an impact on the transcription factor binding site STAT (signal transducer and activator of transcription). Gene expression of *TBX3* could be influenced by the SNP rs8853 and its impact on miR-3189. However an association to breast cancer could not be discovered. Furthermore Douglas and Papaioannou observed *TBX3* overexpression in estrogen-receptor-positive breast cancer cell lines [53]. However, other publications describe an effect of *TBX3* overexpression results in a pool of estrogen receptor negative cancer stem-like cells [56].

*TTN* (Titin or connectin) is the largest polypeptide encoded by the human genome [57] and it has been intensely studied as a component of the muscle contractile machinery [27]. However, *TTN* is expressed in many cell types and has other functions that are compatible with a role in oncogenesis [58–60]. The role of *TTN* as a cancer gene is currently a mathematically based prediction and will require direct biological evaluation. During the present study, 2 out of 6 genotyped SNPs show significant association with increased risk and 4 out of 6 genotyped SNPs with clinical outcome. In addition, more than 50 % of the statistical significant SNPs show an association with negative estrogen or progesterone receptor status. A link between hormones and calcium, which plays a major role in the muscle contractile machinery were Titin is located, could be seen in the estrogen signaling pathway, where the Calcium signaling pathway is a part of. Furthermore, a relation of Calcium signaling pathways and breast cancer is proofed [61, 62].

*ATR* (Ataxia Telangiectasia mutated and Rad3-related), an essential regulator of genomic integrity, controls and coordinates DNA-replication origin firing, replication-fork stability, cell cycle checkpoints, and DNA repair [63]. Smith et al. showed that overexpression of the *ATR* gene resulted in a phenocopy of the i(3q). The genetic alteration of *ATR* leads to loss of differentiation as well as cell cycle abnormalities [64]. Thus *ATR* has been studied as a target for cancer therapy [65]. However new Inhibitors such as caffeine has been proven as fragile and nonspecific [66]. In the present study, rs2227928 was genotyped and statistical analyzed. It is predicted to be tolerated according to Ensembl release [67]. Rs2227928 could be associated with tumour size >2 cm and negative estrogen or progesterone receptor status. It has been frequently studied for an association in different populations. However, they have found no significant differences [68, 69]. These conflicting results about the relationship between rs2227928 and breast cancer could be related to some factors such as sample size and environmental factors but not genetic background. All three populations have European ancestry and can be summarized under the phylogenetic definition Caucasian. In this context, by increasing the sample size number of the French and Finish population an association of rs2227928 and breast cancer could be expected. Some SNPs which are linked with an r^2^ between 85 and 97 to rs2227928 are located in gene *PLS1* (Plastin1). The encoded actin-binding protein has been found at high levels in small intestine [70]. However an association with breast cancer could not be discovered. Regarding signatures of selection rs2227928 shows a significant value among the European vs. African population (Fst =0.076).

Some limitations should be addressed in this study. The statistical power to perform interaction analyses between different SNPs and breast cancer risk is still limited because of our small sample size. In addition, because no data were available on SNP frequencies in any North African population, we used data on the CEU population in our selection process. As also shown by our genotyping, the genetic constitution of the Moroccan population is very similar, and it has been influenced by both European and Sub-Saharan gene flow. However, we may have missed some SNPs private to the North African populations. There may also be some rare SNPs with minor frequency allele or SNPs with still-unknown regulatory properties that were not covered by our study.

## Conclusion

Our preliminary genetic analysis suggests a potential role of germline variations in driver and *APOBEC3* genes in breast cancer susceptibility. These mutations can have impact on clinical outcome and/or BC risk. We could also show that there is a strong association between the polymorphisms in *RUNX1*, *TBX3*, *TTN*, *ATR* genes and the risk of BC. However to verify the results of breast cancer risk and the influence of these polymorphisms further researchers are necessary.

## Abbreviation

BC: breast cancer
OR: odds-ratio
GWASs: genome wide association studies
SNPs: single nucleotide polymorphisms
CNVs: copy number variations
ICGC: International Cancer Genome Consortium
SBR: Scarff-Bloom-Richardson
MAF: minor allele frequency
LD: linkage disequilibrium
UTR: untranslated region
PCR: polymerase chain reactions
HWE: Hardy Weinberg equilibrium
CIs: confidence intervals
*ATR*: Ataxia Telangiectasia mutated and Rad3-related
*STAT*: signal transducer and activator of transcription
*TBX3*: T-box transcription factor 3
